# Conjunctival myxoma masquerading as conjunctival lymphoma: A case report

**DOI:** 10.1016/j.ijscr.2022.107441

**Published:** 2022-07-21

**Authors:** Majed Alkharashi, Hind M. Alkatan, Ahmed A. Alhumidi, Wael Otaif

**Affiliations:** a Department of Ophthalmology, College of Medicine, King Saud University, Riyadh, Saudi Arabia; b King Saud University Medical City, College of Medicine, King Saud University, Riyadh, Saudi Arabia; c Department of Pathology, College of Medicine, King Saud University, Riyadh, Saudi Arabia

**Keywords:** Myxoma, Conjunctiva, Salmon-patch, Lymphoma, Histopathology, Pseudo-elastotic degeneration

## Abstract

**Introduction and importance:**

Ocular myxomas are very rare and can involve the orbit, eyelids, and conjunctiva. Conjunctival myxoma can be misdiagnosed as amelanotic nevus, conjunctival cyst, or ocular surface squamous neoplasia, among others. They can appear as an isolated lesion or can be associated with systemic manifestations as part of the Carney complex or Zollinger-Ellison syndrome.

**Case presentation:**

We describe a 64-year-old healthy male who presented with a right eye painless peri-limbal salmon-colored patch lesion in the infero-temporal bulbar conjunctiva over a period of 2 years. There was no of ocular trauma or surgery and no effect on vision. The mass was not tender, raised, and mobile with fine intrinsic vascularity. Excisional biopsy with the presumed diagnosis of lymphoma revealed a typical sub-conjunctival myxoma.

**Discussion:**

The recognition of ocular myxoma necessitates systemic evaluation to rule out possible associated cardiac myxoma in Carney complex, thus can prevent life-threatening events. The excised mass in our patient showed an area of pseudo-elastotic degeneration, which has further complicated the clinical appearance of the lesion, however, the color, and consistency of the mass were highly suspicious of lymphoma. The diagnosis of myxoma by histopathology was helpful especially in presence of atypical appearance such as in our case.

**Conclusion:**

The histopathological characteristics of conjunctival myxoma can aid in the diagnosis. The lesion in our case was associated with focal severe pseudo-elastotic degeneration and prominent salmon-patch appearing area thus was initially misdiagnosed clinically as a conjunctival lymphoma.

## Highlights

• Conjunctival myxoma is a rare benign tumor that can be easily misdiagnosed.
• Histopathological typical features are essential to confirm the diagnosis.
• We describe a salmon-colored conjunctival myxoma mimicking lymphoma.
• Our case was successfully managed by total excisional biopsy.

## 1 Introduction

Myxoma is a slow-growing benign tumor that originates from the mesenchymal tissue, which may affect the heart, skin, gastrointestinal system, genitourinary system, and musculoskeletal system. Ocular myxomas are extremely rare; however, they can affect the orbit, eyelids, and conjunctiva [1]. Conjunctival myxoma, which is more frequent in older individuals, can be misdiagnosed as amelanotic nevus/melanoma, conjunctival cyst, or ocular surface squamous neoplasia, and lipoma [2], [3]. Typically, they present as painless, fleshy gelatinous masses, and appear to be pink or white. The most common presentation is the cystic form [4]. Ocular myxomas can either present as an isolated lesion or in systemic association, as part of the Carney complex, which is associated with cardiac and cutaneous myxomas, pigmented lesions, and endocrine dysfunction with significant morbidity and mortality, or the Zollinger-Ellison syndrome, which could be associated with pancreatic gastrinoma, inter-atrial septal thickening, and conjunctival myxoma [5]. Therefore, early recognition of an ocular lesion and the initiation of systemic evaluation can prevent life-threatening events.

Here, we describe the clinical presentation and histopathological characteristics of a salmon-patch-like conjunctival myxoma, which was initially misdiagnosed as conjunctival lymphoma due to its clinical appearance. This case report has been prepared and reported in accordance with the SCARE criteria [6].

## 2 Presentation of case

**Fig. 1:**
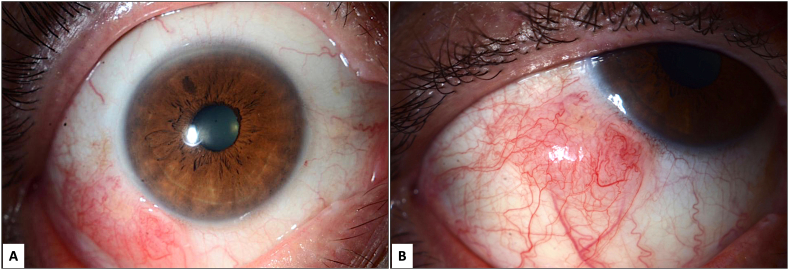
A: Slit lamp clinical image with diffuse illumination showing a raised pink-to-salmon-colored nodular fleshy mass in the inferior temporal part of the bulbar conjunctiva. B: The same peri-limbal mass showing fine intrinsic vascularity. (For interpretation of the references to color in this figure legend, the reader is referred to the web version of this article.)

**Fig. 2:**
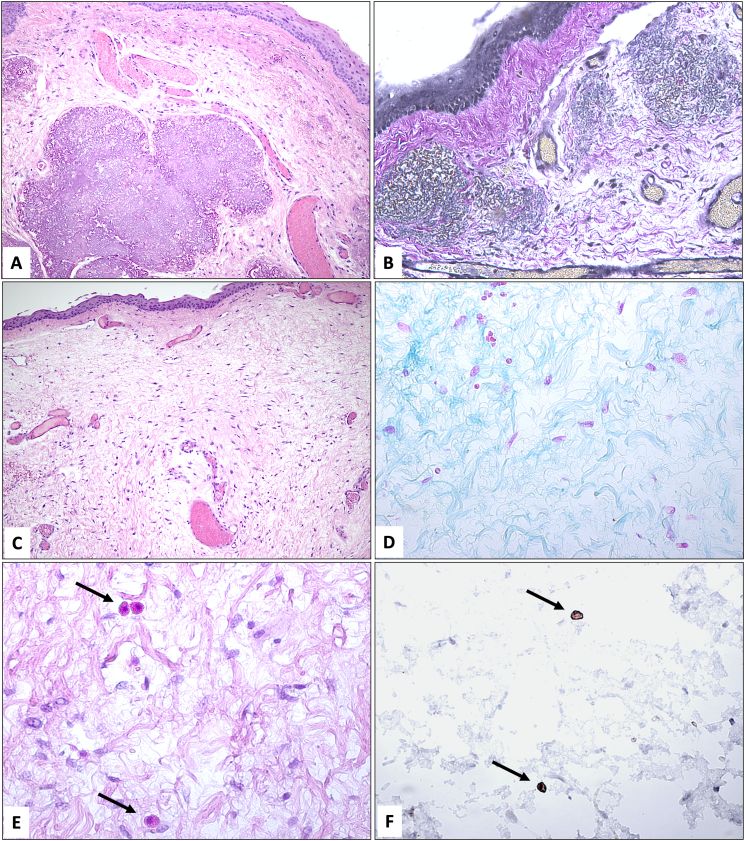
A: Conjunctival epithelium with loss of goblet cells and sub-epithelial area of pseudo-elastotic degeneration (original magnification ×100 Hematoxylin and Eosin). B: Subconjunctival pseudo-elastotic degeneration (original magnification ×200 Elastic stain). C: Histopathological photo of the conjunctival myxoma showing spindle and stellate-shaped cells (original magnification ×100 Hematoxylin and Eosin). D: The subconjunctival myxoma with staining of the myxomatous mucinous background (original magnification ×400 Alcian Blue). E: Another area of the myxoma with stellate-shaped cells and mast cells (Black arrows) (original magnification ×400 Hematoxylin and Eosin). F: The scattered mast cells expressing reactivity with immunohistochemical marker (Black arrows) (original magnification ×400 CD-117). (For interpretation of the references to color in this figure legend, the reader is referred to the web version of this article.)

A 64-year-old healthy male presented to the Ophthalmology department of our tertiary care hospital with ocular discomfort and an infero-temporal peri-limbal painless slow-growing, pinkish nodular lesion involving the bulbar conjunctiva of the right eye (RE), which has been present over the last 2 years. The patient didn't report any history of ocular trauma or surgery. His family history, drug history, and medical history were unremarkable. The uncorrected visual acuity measured 20/30 in both eyes. Slit lamp biomicroscopic examination of the left eye was normal, while that of the RE showed a non-tender, pinkish, mobile, raised lesion on the temporal part of the bulbar conjunctiva, measuring 9 mm × 7 mm in diameter with fine intrinsic vascularity (Fig. 1: a & b). Gonioscopic and dilated fundus examination were both normal. There was no palpable lymphadenopathy. The clinical impression was conjunctival lymphoma. This was discussed with the patient, who agreed on undergoing excisional biopsy of the mass under local anesthesia owing to the clinical suspicion of lymphoma. The procedure was done by a specialized corneal surgeon, who began with marking the margin of the lesion, followed by a subconjunctival injection of 2 % lidocaine and 1:100,000 epinephrine. Dissection of the adjacent conjunctiva to excise the lesion “en toto” with a 4 mm safety margin was performed. During the dissection there was a clear plane between the tumor and the underneath sclera. Finally, the conjunctiva was closed with 8-0 interrupted vicryl sutures. The patient tolerated the procedure well and there were no reported complications. Postoperatively, 1 % drop of prednisolone acetate (Pred Forte; Allergan, Inc., Irvine, CA) was prescribed with the tapering dose for a period of four weeks, in conjunction with topical 0.05 % moxifloxacin (Vigamox; Alcon Laboratories, Inc., Fort Worth, TX) for two weeks. Subsequent follow-up showed no recurrence of the lesion over a period of 2 months. Histopathological examination showed irregular elevated conjunctival non-keratinizing stratified squamous epithelium with loss of goblet cells. The underlying substantia propria showed in one focal area dense collections of fragmented curly fibers representing pseudo-elastotic degeneration highlighted using Elastic stain (Fig. 2a & b) and surrounding larger hypocellular area of proliferating spindle and stellate-shaped cells within loose myxoid stroma that showed diffuse positive staining with Alcian blue and colloidal iron stains (Fig. 2c & d). The stroma also showed fine blood vessels and wavy thin collagen fibers. Several mast cells were also present with scattered positivity with CD117 (Fig. 2e & f). The diagnosis of conjunctival myxoma was confirmed. Based on the final histopathological diagnosis and the known possible associations with myxoma, further systemic evaluation was conducted and similar myxomas in other locations of his body were excluded. There were no associated systemic conditions.

## 3 Discussion

Conjunctival myxomas are extremely rare tumors, with a rate of incidence of 0.001–0.002 %, as compared to other conjunctival tumors. They usually appear in older individuals, as in our present case [1]. However, it could also appear in younger patients [3].

Normally, conjunctival myxoma tends to present as a painless, slow-growing, translucent, yellow-pinkish conjunctival mass that can be cystic, and according to the literature, it is more frequently present in the bulbar conjunctiva, being characteristic in the temporal quadrant as in our case [2], [7], [8]. Nonetheless, the conjunctival lesion in the present patient had an atypical clinical presentation, which was more suspicious of a lymphoma with a salmon-colored nodular fleshy patch involving the bulbar conjunctiva [9].

Although most conjunctival myxomas reported in the literature present as isolated lesions, it is worth mentioning that some of them could appear as a part of the Carney complex or the Zollinger-Ellison syndrome. Therefore, a clinical suspicion of such conjunctival lesion mandates taking into account atypical forms, and early recognition if possible with an excisional biopsy and an initiation of systemic evaluation is advisable in order to prevent life-threatening events since ocular presentation may precede the embolic vascular events, especially in the previously mentioned syndromes [1], [10]. Histopathological analysis is essential for the diagnosis of this condition [1]. This tumor is typically composed of spindle and stellate cells immersed in an abundant mucinous matrix, with few vessels and reticulin fibers [3]. This fact was supported by Stout in 1948 who found that myxomas are a true tumor consisting of stellate-shaped cells embedded in a loose myxoid stroma through which delicate reticulin fibers pass in various directions [1], [11]. Multinucleated and vacuolated cells with slightly pleomorphic nuclei and floret-type giant cells were also described according to the latest systematic review [1]. Scattered lymphocytes, macrophages, and mast cells have been also reported in myxomas [2]. Mast cells were observed in our case and were highlighted by expression of reactivity to CD117.

Immunohistochemistry is characterized by positivity for vimentin and negativity for smooth muscle actin, SOX10, and GLUT1 [3]. Mucoid material stains positive for Alcian blue, as was seen in our case [3]. Other auxiliary tests reported in the literature which can help diagnose conjunctival myxomas are orbital ultrasonography, ultrasound biomicroscopy, and orbital magnetic resonance [12], [13]. Nonetheless, these scans showed different patterns causing some confusion. Therefore, a histological analysis from an excisional biopsy is still mandatory [3].

Regarding management, excisional biopsy seems to be the preferred pattern, according to the results from a review by Alvarado-Villacorta et al. [3]. This procedure reported a good prognosis with a low risk of recurrence. Recurrence after surgical excision has been reported even after long periods of follow up including one recurrence in association with Carney complex [10], [11]. Moreover, no malignant transformation has been reported in this tumor [14]. The present case supports this behavior where excisional biopsy was originally performed to rule out conjunctival lymphoma with no recurrence since the excision. The histopathological analysis helped us obtain the correct diagnosis.

## 4 Conclusion

Conjunctival myxomas are a rare condition which can mimic other benign and malignant ocular lesions. Surgical excision is required to confirm the diagnosis and to exclude other malignancies. Furthermore, these lesions can be associated with systemic syndromes; therefore, an early recognition and initiation of systemic evaluation is necessary to prevent life-threatening events. We would like to highlight the possible atypical appearance as well of conjunctival myxomas. Therefore, myxoma should be always considered within the differential diagnosis of a conjunctival lesion.

## Provenance and peer review

Not commissioned, externally peer reviewed.

## Sources of funding

This research received no specific grant from any funding agency in the public, commercial or not-for-profit sectors.

## Ethical approval

IRB is not required for case reports. However, information was obtained and reported in a manner that was compliant with the standards set forth by the Health Insurance Portability and Accountability Act, and the Declaration of Helsinki as amended in 2013.

## Consent

General informed written consent was obtained from the patient including permission for anonymous use of photos and for reporting. A copy of the written consent is available for review by the Editor of this journal upon request.

## Author contribution

• Majed Al-Kharashi: The primary treating ophthalmic surgeon providing clinical images.
• Wael Otaif: Chart review for data collection, literature review and first draft of the case report.
• Ahmed Alhumidi: Histopathological examination as consultation.
• Hind M. Alkatan: Final tissue diagnosis and images. Critical overall review and revision of the manuscript for submission as Corresponding author.

## Research registration

Not applicable.

## Guarantor

Hind M. Alkatan.

## Declaration of competing interest

None.
